# Monogenic Autoinflammatory Diseases: State of the Art and Future Perspectives

**DOI:** 10.3390/ijms22126360

**Published:** 2021-06-14

**Authors:** Giulia Di Donato, Debora Mariarita d’Angelo, Luciana Breda, Francesco Chiarelli

**Affiliations:** Department of Pediatrics, University of Chieti, 66100 Chieti, Italy; didonatogiuls@gmail.com (G.D.D.); deboramrdangelo@gmail.com (D.M.d.); luciana.bredach@gmail.com (L.B.)

**Keywords:** systemic autoinflammatory diseases, inflammasomopathies, interferonopathies, periodic fever, next generation sequencing

## Abstract

Systemic autoinflammatory diseases are a heterogeneous family of disorders characterized by a dysregulation of the innate immune system, in which sterile inflammation primarily develops through antigen-independent hyperactivation of immune pathways. In most cases, they have a strong genetic background, with mutations in single genes involved in inflammation. Therefore, they can derive from different pathogenic mechanisms at any level, such as dysregulated inflammasome-mediated production of cytokines, intracellular stress, defective regulatory pathways, altered protein folding, enhanced NF-kappaB signalling, ubiquitination disorders, interferon pathway upregulation and complement activation. Since the discover of pathogenic mutations of the pyrin-encoding gene MEFV in Familial Mediterranean Fever, more than 50 monogenic autoinflammatory diseases have been discovered thanks to the advances in genetic sequencing: the advent of new genetic analysis techniques and the discovery of genes involved in autoinflammatory diseases have allowed a better understanding of the underlying innate immunologic pathways and pathogenetic mechanisms, thus opening new perspectives in targeted therapies. Moreover, this field of research has become of great interest, since more than a hundred clinical trials for autoinflammatory diseases are currently active or recently concluded, allowing us to hope for considerable acquisitions for the next few years. General paediatricians need to be aware of the importance of this group of diseases and they should consider autoinflammatory diseases in patients with clinical hallmarks, in order to guide further examinations and refer the patient to a specialist rheumatologist. Here we resume the pathogenesis, clinical aspects and diagnosis of the most important autoinflammatory diseases in children.

## 1. Background

It is known by now that an early inflammatory phenotype plays a primary pathophysiological role in an expanding group of syndromes of the paediatric age. Systemic autoinflammatory diseases (SAIDs) are a heterogeneous family of disorders characterized by a dysregulation of the innate immune system, with antigen-independent immune pathways hyperactivation and development of sterile inflammation [1]. They mainly present in infancy or childhood with recurrent episodes of fever, laboratory signs of systemic inflammation and various symptoms involving joints, skin, serosal membranes, gastrointestinal (GI) tube, central nervous system (CNS) and other tissues. Given the wide clinical variability and rarity of these conditions, they are often misunderstood, with delayed diagnosis and treatment. SAIDs were first recognized as distinct clinical entities nearly 20 years ago [2], in opposition to autoimmune diseases, due to the lack of circulating autoantibodies and adaptive immune system involvement: the term “autoinflammatory” describes the onset of unprovoked inflammation, caused by an excessive production of proinflammatory cytokines or by a defective shutdown of inflammatory responses, in the absence of autoreactive or antigen-specific T-cells [2]. Since the understanding that Familial Mediterranean Fever (FMF), the most frequent inherited inflammatory disease, derives from mutations in the pyrin-encoding gene [3], more than 50 new monogenic SAIDs havebeen discovered thanks to the advances in genetic sequencing [4]. In most cases, AIDs have a strong genetic background, with mutations in single genes: monogenic AIDs arise from loss-of-function mutations in pro-inflammatory genes and/or gain-of-function mutations in genes that stimulate inflammation. Therefore, they can derive from different pathogenic mechanisms at any level, such as dysregulated inflammasome-mediated production of cytokines, intracellular stress, defective regulatory pathways, altered protein folding, enhanced nuclear factor-kβ (NF-κB) signalling, ubiquitination disorders, interferon (IFN) overproduction and complement activation [5]. On the other hand, SAIDs can also be of polygenic or multifactorial origin, with epigenetic and environmental factors influencing the phenotype [6]. Over the past two decades, the discovery of genes involved in human AIDs has allowed a better understanding of the underlying innate immunologic pathways and pathogenetic mechanisms, thus opening new perspectives in targeted therapies [7]. Moreover, the advent of next generation sequencing (NGS) has increased this field of research, which has become of great interest [7].

## 2. The Innate Immune System and Inflammatory Response

Innate immunity is an evolutionarily conserved system representing the first line of defence against foreign microbes and self-danger signals, allowing for recognition and elimination of pathogens and infected cells, recruitment of the specific adaptive system, maintenance of self-tolerance and tissue repair [8]. The innate immune system acts through the interaction of four components: the epithelial barrier, the cellular compartment (neutrophils, macrophages, mast cells, natural killer lymphocytes (NKs), plasma defensive proteins (for example complement, reactive C protein (RCP)) and cytokines. The first step of the inflammatory response is recognition, since phagocytic cells can identify highly conserved structures on the pathogen’s surface, such as viral RNA or bacterial lipopolysaccharide (pathogen-associated molecular patterns (PAMPs)), and endogenous molecules released by damaged cells, called damage-associated molecular patterns (DAMPs). These molecules are recognized by pattern recognition receptors (PRRs) of phagocytes, leading to the transcription of proinflammatory genes, the production of cytokines and chemokines and the induction of a proinflammatory cell death, called pyroptosis. PRRs include Toll-like receptors (TLRs), C-type lectin receptors (CLRs), stimulator of interferon genes (STING), and some intracellular sensors, including nucleotide-binding oligomerization domain (NOD)-like receptors (NLRs), Absence in melanoma 2 (AIM2)-like receptors (ALRs) and pyrin [9]. Inflammasomes are a family of protein complexes made of different sensor and adapter molecules (apoptosis-related speck-like protein containing caspase activation and recruitment domains (ASC)) whose interaction stimulate the activation of caspase-1, leading to the production of active pro-inflammatory cytokines interleukin 1 β (IL-1β) and interleukin-18 (IL-18) and cleavage of gasdermin-D (GSDMD). The N-terminal domain of GSDMD (GSDMD-N) forms cytotoxic pores in the lipidic cellular membrane, causing pyroptosis [10]. However, some NLRs mediate caspase-independent nuclear NF-κB and mitogen-activated protein kinase (MAPK) signalling. Also, S100 proteins are sensed by TLRs (mainly TLR-4) and activate NF-κB pathway, enhancing NLR family pyrin domain containing (NLRP) 3 (NLRP-3) transcription [11]. Simultaneously, the Interferon (IFN) system, through a partially differentpathway, represents yet another powerful pathway of innate immunity capable of interfering with viral antigens and certain bacterial infections. Precisely, type I IFNs are powerful inflammatory polypeptides, that are ubiquitously expressed by every immune and non-immune cell and can be induced mostly by microbial and viral nucleic acids [12,13]. The accumulation of nucleic acids during viral replication is sensed in the cytoplasm or endosomes by two systems: (1) double-strand DNA (dsDNA) activates the cytoplasmic DNA receptors cyclic GMP-AMP synthase (cGAS) that fire the STING in endoplasmic reticulum (ER), and then, IFN regulatory factors (IRF) 3 and 7; (2) dsRNA activate the cytosolic RNA helicases retinoic acid-inducible gene I (RIG-I)—melanoma differentiation-associated protein 5 (MDA5) system, which leads the formation of the mitochondrial antiviral signalling (MAVS) signalling complex and activates also IRF3 and IRF7. Finally, IRF3 and IRF7 translocate to the nucleus and induce the transcription of IFN. Type I IFNs bind to IFN receptors (IFNR) through autocrine and paracrine action [14,15,16,17,18]. The IFNR dimerization activates signal translation through the Janus kinase/signal transducers and activators of transcription (JAK-STAT) pathway, promoting the expression of interferon-stimulated genes (ISGs), stopping cell replication and protein translation of the infected cell and stimulating the immune response against pathogen antigens [14,15,19]. At the same time, a negative feedback mechanism comes from the ubiquitin-specific protease 18 (USP18)/ISG15 system. The USP18 binds the IFNAR2 subunit, decoupling it from JAK1 and inhibiting the propagation of the next signal. ISG15 prevents the degradation of USP18 by sphingosine kinase 2 (SPK2) [20,21]. Finally, several recent studies reported an essential role of type I IFNs in non-canonical NLRP3 inflammasome activation and pyroptosis. Different levels of regulation are involved in the cross talk of IFNs in inflammasome, highlighting a fascinating interaction between these 2 inflammatory systems, that could be a future frontier for research, in the context of AIDs [22].

**Figure 1:**
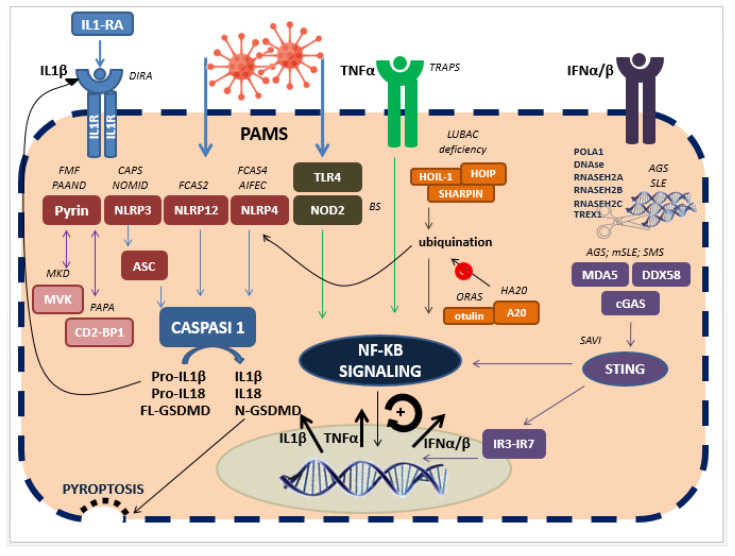
Inflammatory signalling pathways in AIDs. The first step of inflammatory response is the recognition of pathogens’ conserved structures (PAMP) by PPR of intracellular sensors (including NLR domains, ALR and pyrine), with the formation of a multimeric proteic complex, called inflammasome. The inflammasome receptors interact with the adapter protein ASC, leading to the activation of Caspase 1, which converts pro-IL-1β and pro-IL-18 to their bioactive forms. Caspase 1 also cleavages the Gasdermin-D (GSDMD), whose N-terminal domain (GSDMD-N) forms cytotoxic pores in the lipidic cellular membrane, causing pyroptosis. Also, PAMPs are sensed by TLRs (mainly TLR-4) or NOD2 receptors and activate NF-κB pathway, enhancing NLRP-3 transcription. The intra-cytoplasmic accumulation of viral or endogenous nucleic acids is sensed by others proteins, like cGAS, MDA5 and DDX58, which activate STING and therefore IR3-IR7 factors. These latter translocate to the nucleus, stimulating the transcription of type I IFN genes. STING also directly activates NFkB signalling. Most of the described signalling pathways activate NF-κB signalling, thus stimulating the transcription of NF-κB-dependent genes (NLRP3, pro-IL-β, pro-IL-18, and IL-6) and promoting a positive feedback effect. Alterations at different levels of this complex mechanism are associated with the development different AIDs. *Abbreviations*: PAMP (pathogen associated molecular patterns); PRR (pattern recognition receptors); NLR (Nucleotide-binding oligomerization domain (NOD)-like receptors); ALR (Absence in melanoma 2 (AIM2)-like receptors); NLRP-3 (NLR family pyrin domain containing 3); NF-κB (NF-kappaB); IFN (interferon); IRF (IFN regulatory factor); CGAS (cyclic GMP-AMP synthase); MDA5 (melanoma differentiation-associated protein 5; DDX58 (DExD/H box RNA helicase); STING (Stimulator of interferon genes).

The most important AIDs can be classified according to the predominance mechanism of inflammation into inflammasomopathies, or IL-1β-related syndromes, NF-κB activation disorders, cytokine-signalling disorders and interferonopathies. The most relevant AIDs, their genetic and pathologic background and their phenotypes aredescribed in Table 1. Figure 1 summarizes the main inflammatory signalling pathways involved in AIDs.

## 3. Inflammasomopathies

Numerous inflammasomes have been identified and defined by their nucleating proteins, including pyrin, NLRP1, NLRP2, NLRP3, AIM2 and NLR Family CARD Domain Containing (NLRC) 4 (NLRC4). Mutations of these molecules and/or of inflammation regulatory genes lead to inappropriate inflammasome activation, reported in many childhood rheumatic diseases. Clinical differences among the inflammasomopathies reflect the severity of genetic defects and the presence of mosaicism, as well as the cellular distribution of a particular inflammasome and its function. For example, pyrin and NLRP3 are widely expressed in innate immune cells and activate pro-IL-1β [23], while NLRC4 is expressed in the gut and has pro-IL-18 as substrate, contributing to the development of severe enterocolitis and macrophage activation syndrome (MAS) [24]. Finally, NLRP1 genetic variants have been associated with skin involvement [25].

### 3.1. Pyrin Inflammasomopathies

#### 3.1.1. Familial Mediterranean Fever (FMF)

FMF is the most common monogenic AID, with the highest prevalence in the Mediterranean areas, especially among Armenians, Turks, Arabs, Sephardic Jews, Italians and Greeks. It is caused by autosomal recessive (AR) mutations of *MEFV* gene located on chromosome 16 and encoding for the protein pyrin. The important number of *MEFV* heterozygotes among Ashkenazi Jews and Turks (>20%) explains the pseudodominant mode of inheritance in these populations [26]. These genetic variants seem to confer enhanced resistance to *Yersina pestis* and other microbes which are able to neutralize the conventional pyrin inflammasome assembly [27].

Pyrin inflammasome does not directly interact with PAMPs and DAMPs; precisely, several bacterial virulence factors enhance pyrin activation through a negative modulation of Ras Homolog Family Member A (RhoA) GTPase. This molecule activates two serine-threonine kinases (PKN1 and PKN2) which bind and phosphorylate pyrin: the interaction between phosphorylated pyrin and the regulatory protein 14-3-3 blocks pyrin inflammasome activity. Gain-of-function mutations that primarily occur in the B30.2 domain confer poor affinity to PKN1, PKN2 and 14-3-3, leading to constitutive activation of pyrin inflammasome [28]. A mutation in exon 2 of the *MEFV* gene (c.726C > G; p.Ser242Arg) is associated with an autosomal dominant (AD) syndrome characterized by early onset of periodic fever, neutrophilic dermatosis, severe acne, pyoderma gangrenosum, sterile cutaneous abscesses and arthromyalgia, called **pyrin-associated auto-inflammation with neutrophilic dermatosis (PAAND)**. This mutation interferes with 14-3-3 function, inducing inflammasome hyperactivation [29]. PAAND has been successfully treated with anti-IL1 Anakinra [30].

FMF onset usually occurs in childhood, before 5 years of age, with recurrent episodes of fever, serositis and elevated inflammatory markers, lasting 12–72 h. Delayed presentation has also been described: it is associated with attenuated disease course and good response to colchicine [31]. Patients can present with peritonitic abdominal pain, pleuritis, non-erosive acute arthritis of large joints and erysipela-like lower extremity rash; associated pericarditis and orchitis are less frequent [32]. Attacks can be triggered by stress, infections, even drugs (metaraminol, cisplatin) and resolve spontaneously, with clinical well-being during free intervals [4]. The most important complication of FMF is secondary amyloidosis and eventually end-stage renal disease. Persistent inflammation can be found in 30% of children between the attacks, influencing growth and bone health, and some asymptomatic carriers could have a constant elevation of serum amyloid A (SAA), with the risk of developing amyloidosis over time. This risk is higher in patients with M694V mutation (c.2080_2082delinsGTA; p.Met694Val) and SAA1 allele [33]. Colchicine is the main treatment of FMF, which decreases attack frequency and prevents secondary amyloidosis. It activates RhoA and causes microtubule destabilization, thus inhibiting pyrin inflammasome activation [34]. Anti-IL1 are effective in patients who are unresponsive or intolerant of colchicine [35]. Also, tumor necrosis factor (TNF)-inhibitors have been used in colchicine-resistant patients, especially with articular involvement, with good responses reported in observational studies [36]. Different diagnostic criteria have been proposed for FMF over time, from Tel Hashomer Criteria in 1967 [37] to the Turkish FMF paediatric Criteria in 2009 [38]. However, the validation of these criteria in other ethnic groups and in more genetically heterogeneous populations is still limited and a definitive diagnosis of FMF continues to be a challenge. In this set, genetic confirmation is useful to support the diagnosis. So far, more than 340 *MEFV* sequence variants have been reported and many of those have no clear pathogenic role, so that a careful interpretation of genetic sequencing is mandatory [39]. The most frequent pathogenetic variants are located on exon 10 (c.2080_2082delinsGTA- p.Met694Val; c.2040G > T-p.Met680Ile; c.2177T > C-p.Val726Ala; c.2282G > A-p.Arg761His; M649I and A744S), the firstbeing associated with the most severe phenotype and early onset, both in homozygosity and compound heterozygosity [40]. E148Q in exon 2 is one of the most frequent sequence alterations in the *MEFV* gene, but it is also frequent in the general population (up to 30% in Asia) and its pathogenic role remains uncertain [40]. With the increasing availability of genetic testing, many patients with recurrent fever have been found to carry a heterozygous mutation of *MEFV* with a consistent risk of AA amyloidosis and a good response to colchicine treatment. On the other hand, clinical variability can be explained by the combination of heterozygous *MEFV* mutations and mutations of other monogenic AIDs [41]. Finally, an AD pattern of transmission has been reported [42]. Recent evidence has better clarified that FMF is secondary to gain-of-function mutations of *MEFV* with a dose-dependent effect, so that FMF can be diagnosed in the presence of a consistent clinical phenotype associated with either one or two pathogenic variants (heterozygous in AD disease, homozygous or compound heterozygous in AR disease). Even compound heterozygous for one pathogenic and one non-pathogenic *MEFV* variant, heterozygous for one pathogenic variant or biallelic non-pathogenic mutations should be considered in the diagnostic process [43]. The presence of pathogenic mutations, even in paucisymptomatic patients, needs a close follow up and eventual treatment to prevent complications [40]. The new Eurofever/PRINTO Classification Criteria [43] are resumed in Table 2.

#### 3.1.2. Mevalonate Kinase Deficiency (MKD)

MKD is a rare AR disease, first described in Western Europe and known as **Hyper-IgD Syndrome (HIDS)**, because it is associated with the increase of serum Immunoglobulin D (IgD) in many patients. It is caused by loss of function mutations in the gene encoding for mevalonate kinase (*MVK*) on chromosome 12 [44]. According to the residual enzyme activity, the clinical spectrum varies from MKD/HIDS, with predominant autoinflammatory symptoms and residual enzymatic activity ranging between 1.8 and 28% of normal values, to mevalonic aciduria (MA), with no remaining enzymatic function, recurrent fever, growth failure, dysmorphia and severe neurological involvement [45]. MKD usually appears within the first six months of life, with recurrent inflammatory attacks, characterized by fever lasting 3–7 days associated with gastrointestinal symptoms (abdominal pain, diarrhoea, vomiting), arthromyalgia or arthritis, cutaneous involvement (non-migratory maculo-papular or urticarial rash), aphthous stomatitis, hepatosplenomegaly, painful cervical adenopathy and headache. These episodes can be triggered by vaccinations, stress and infections and may relapse every 4–8 weeks [46]. MKD is rarely complicated by AA amyloidosis, except for patients carrying V377I/I268T genotype (c.1129G > A-p.Val377Ile/c.803T > C-p.Ile268Thr) [47]. Another concern is the possible development of MAS. The presence of elevated IgD levels both in-between attacks is not specific, since it has also been reported in FMF and TNF receptor-associated periodic syndrome (TRAPS); furthermore, 20% of patients with MKD show normal IgD levels and IgD value is not related to disease severity [48]. If MKD is suspected, dosage of urinary mevalonic acid is a reliable diagnostic tool [49]. MVK is an enzyme of the cholesterol and isoprenoid pathway. Its loss of function reduces the prenylation of proteins, such as geranylgeranyl pyrophosphatase, which is necessary for Rho-A activation and phosphatidyl-inositol 3 kinase (PI3K)-mediated inhibition of pyrin inflammasome [50]. This mechanism suggests an interesting molecular connection between FMF and MKD. Nevertheless, unlike FMF, colchicine is not very effective in inhibiting pyrin in MKD patients, probably because it cannot activate RhoA that is not localized to cell membrane through geranylgeranylation [28]. On the other hand, a role of impaired autophagy, altered antioxidant response and mitochondrial dysfunction has also been described [45]. More than 210 variants of *MVK* gene have been identified so far; the most frequent mutation is V377I either in homozygous or as a compound heterozygous with I268T [47]. Recently, Carapito R. et al. reported the case of two sisters with the same mutation (V377I/V377I) but showing different clinical manifestations: one sister was asymptomatic and the other was symptomatic due to the presence of a gain of function mutation of *STAT1* gene (c.722G > A-p.Arg241Gln), influencing the phenotype [51]. This finding could provide therapeutic options through inhibition of the JAK/STAT pathway [52].

#### 3.1.3. Other Pyrin-Associated Inflammasomopathies

The expansion of genetic analyses application is paving the way for the discovery of new monogenic disorders, characterized by recurrent multiorgan symptoms, rather than prominent fever. **Pyogenic arthritis, pyoderma gangrenosum and acne (PAPA)** is an AD disease, caused by gain-of-function mutations of proline-serine-threonine phosphatase interacting protein 1 (*PSTPIP1*) gene, located on chromosome 15 [53]. This gene encodes for CD2-binding protein 1 (CD2-BP1), a regulatory protein which interacts with pyrin and enhances pyrin inflammasome activation; it is also involved in cytoskeletal organisation and white cell migration. Furthermore, the increased release of the alarmins myeloid-related protein (MRP) 8 and 14 by activated phagocytes and keratinocytes stimulates TLR4-mediated immune responses and exerts a positive feed-back mechanism with IL-1β [54]. PAPA is characterized by skin and joint involvement. The latter usually appears in early childhood in the form of a sterile oligoarthritis and tends to regress over time, while skin lesions, such as severe cystic acne, pyoderma gangrenosum, aseptic abscesses and oral ulcers, typically present during puberty and persist [55]. Some patients present pyoderma gangrenosum with or without hidradenitis suppurativa, a particular clinical entity named **pyoderma gangrenosum, acne and hidradenitis suppurativa (PASH syndrome)** [56]. Furthermore, particular *PSTPIP1* mutations, such as E250K (c.748G > A-p.Glu250Lys) have been associated with a distinct clinical entity, called **PSTPIP1-associated myeloid-related-proteinemia inflammatory syndrome (PAMI)** and characterized by PAPA-like symptoms, hepatosplenomegaly, pancytopenia and growth restriction. These mutations cause a charge reversal in *PSTPIP1* y-domain and increase interaction with pyrin [57]. Recently, Belelli et al. described the case of a patient with a heterozygous E250K mutation showing a mild clinical phenotype with recurrent knee monoarthritis, bone marrow involvement and no skin lesions. In this patient, anti-IL1 Anakinra was effective against systemic inflammation, without controlling cytopenia [58]. Given the large clinical spectrum, the comprehensive term ***PSTPIP1*-associated AIDs** has been proposed. Finally, WD repeat-containing protein 1 (WDR1), a protein involved in actin depolymerization, also regulates pyrin inflammasome. A missense mutation of its encoding gene causes actin accumulation, pyrin activation and IL-18 overproduction and it has been associated with a new clinical entity **(Periodic Fever Immunodeficiency and Thrombocytopenia or PFIT)**. Unlike traditional therapies, hematopoietic stem cell transplantation (HSCT) seems to be effective in these patients [59].

### 3.2. NLRP3 Inflammasomopathies

NLRP3 inflammasome can be activated by different stimuli. PAMPs, DAMPs and S100 proteins, released by activated granulocytes and monocytes, bind to TLRs, mainly TLR4, and activate NF-κB pathway, thus stimulating NLRP3, pro-IL1β and pro-IL18 transcription. Important second stimulating signals for NLRP3 inflammasome aggregation include potassium efflux, changing in intracellular calcium levels, reactive oxygen species (ROS), mitochondrial damage, lysosomal rupture with cathepsin B release, lipid particles and crystal such as uric acid [60]. Therefore, many studies showed NLRP3 pathway’s implication in many acquired diseases, such as gout [61], type 2 diabetes [62] and atherosclerosis [63].

#### Cryopyrin-Associated Periodic Syndromes (CAPS)

The term CAPS, or **NLRP3-associated AID (NLRP3-AID)** [64] describes a continuum of clinical entities of increasing severity, whose pathogenetic mechanism is a gain-of-function mutation of NLRP3 gene, also known as cold-induced autoinflammatory syndrome 1 (*CIAS1*) gene, on chromosome 1q44, encoding for cryopyrin [65]. Hyperactivated cryopyrin enhances caspase-1 activity, leading to overproduction of activated IL-1β [66]. CAPS displays a chronic or acute intermittent course, according to disease severity, with almost persistent elevation of inflammatory markers. Three clinical syndromes have been described [67]. Patients with **familial cold autoinflammatory syndrome (FCAS)** present with cold-triggered recurrent episodes of fever, urticaria-like rash, conjunctivitis and arthralgia. **Muckle-Wells syndrome (MWS)** has a more chronic than recurrent pattern, and it is characterized by the association of urticaria and sensorineural hearing loss, with an increased risk of AA amyloidosis (25% of patients). Finally, **chronic infantile neurologic cutaneous articular (CINCA) syndrome/neonatal onset multisystem inflammatory disease (NOMID)** manifests with the onset of neurological, cutaneous and articular involvement in the first days of life. Patients present with diffuse non-pruritic urticarial rash, chronic aseptic meningitis, progressive deforming arthropathy of inferior limbs, skeletal abnormalities with epiphyseal overgrowth and a characteristic facial dysmorphia, with frontal bossing and saddle-nose deformity [68,69]. CAPS is caused either by AD inherited germline or de novo mutations of NOD domain of *NLRP3* gene [70]. More than 200 sequence variants have been identified so far, mainly in the exon 3, with a strict genotype-phenotype correlation. For example, neurological involvement is rare in patients carrying A439V (c.1322C > T-p.Ala441Val), V198M (c.598G > A-p.Val200Met) and E311K (c.931G > A-p.Glu311Lys) mutations, while children with T348M variant (c.1049C > T (p.Thr350Met) show an early-onset phenotype with hearing loss [70]. Interestingly, traditional techniques of genetic analysis do not always allow for the identification of clear pathogenic mutations in patients with CAPS-like symptoms, due to the presence of mosaicism or unknown epigenetic factors [71]. Furthermore, somatic mutations have been identified in 0.5–19% of patients with CAPS, especially with more severe forms [72]. On the other hand, CAPS-like phenotypes can be associated with mutations of other related genes, such as *NLRP12*, *NLRC4* and *Factor12* [73]. For example, *NLRC4* gene mutations have been associated with **familial cold autoinflammatory syndrome 4 (FCAS4)**, characterized by neonatal-onset, cold-induced urticarial rash and arthralgia [74]. Furthermore, heterozygous *NLRP12* mutations have been reported in patients presenting with recurrent episodes of fever, arthralgia and cold-induced urticaria (**familial cold autoinflammatory syndrome 2 FCAS2**). The observed genetic variants altered NLRP12 constitutive inhibition of NF-κB [75]. Therefore, a deep genetic analysis with NGS is mandatory for genetic confirmation of CAPS [76], in addition to the research of clearly pathogenic variants, such asR260W (c.784C > T-p.Arg262Trp), D303N (c.913G > A-p.Asp305Asn), L305P (c.920T > C-p.Leu307Pro), E311K, T348M (c.1049C > T-p.Thr350Met), L353P (c.1064T > C-p.Leu355Pro), A439V and V198M [39]. The new Eurofever/PRINTO Classification Criteria for CAPS [43] are resumed in Table 3.

## 4. Disorders of TNF/NF-κB Activity

TNF and NF-κB are closely related: TNF family receptors are important activators of NF-kB pathway, whose function also results in TNF production [77]. Regulation of NF-κB pathway is very complex and based on sensor proteins, ubiquitin- modifications and inhibitory proteins [78]. Alterations at any level of this pathway can lead to a group of AID characterized by systemic inflammation, fever and granulomas formation [79]. These clinical entities often partially and temporarily respond to therapeutic TNF inhibition.

### 4.1. TNF Receptor-Associated Periodic Fever Syndrome (TRAPS)

TRAPS is an AD disease due to mutations of the TNF receptor superfamily member 1A (*TNFRSF1A*) on chromosome 12 encoding for the 55-KF receptor of TNF-α [2]. It is characterized by recurrent inflammatory attacks, lasting from 5 days to 3 weeks, even though some patients can present with a chronic course with exacerbations. The attacks can be triggered by emotional stress, infections, vaccinations, exercise and menstrual cycle. A periorbital oedema and pseudo-cellulitis rash of the limbs and chest are hallmarks of the disease. Other signs and symptoms include elevated inflammatory markers, fever, leucocytosis, abdominal pain, migrating myalgia, arthralgia, chest pain, testicular pain, conjunctivitis, lymphadenopathy and polymorphic skin lesions, including urticaria. The mean age at presentation is 4.3 years, but symptoms can also appear later in life [80]. AA amyloidosis has been described in 18% of patients [81]. 158 *TNFRSF1A* gene sequence variations have been identified so far, mainly in exon 2, 3 and 4. The two most common *TNFRSF1A* variants (c.224C > T-p.Pro75Leu) and c.362G > A-p.Arg121Gln) are of low penetrance and have also been identified in 10% of healthy west Africans and 2% of healthy Caucasians respectively; these variants have an unclear role and often result in mild disease, with shorter and/or more frequent fever episodes [82,83]. Somatic mosaicism has also been reported [84]. The majority of pathogenic mutations of *TNFRSF1A* are missense substitutions altering cysteine-cysteine disulphide bonds in the extracellular receptor domain, which are important for protein folding [80]. The result is altered receptor trafficking, impaired receptor clearance with accumulation in the ER, increased ROS release, NF-κB and MAPK pathways activation [85]. On the other hand, defective autophagy of TNFR1 aggregates seems to trigger innate immunity with NLRP3 inflammasome activation and cytokines (IL-1β) overproduction [86]. In the absence of confirmatory genetic testing, the diagnosis relies on clinical judgement. The new Eurofever/PRINTO Classification Criteria for TRAPS [43] are outlined in Table 4.

### 4.2. Relopathies

NF-κBopathies are called Relopathies, because RelA and RelB are key components of the NF-κB complex. **Blau syndrome (BS)** is an AD disease associated with genetic variants of *NOD2* or caspase recruitment domain containing protein 15 (*CARD15*) [87]. Somatic mosaicism has been reported in BS, similar to CAPS and TRAPS [88]. Besides, de novo mutations of these genes have been linked to early onset sarcoidosis [89]. NOD2 is a cytosolic member of the NLR family, whose binding to bacterial muramyl-dipeptide stimulates self-oligomerization, receptor-interacting serine/threonine-protein kinase 2 (RIPK2) recruitment and activation of NF-κB and MAPK pathways. Gain-of-function mutations in BS alter autoinhibitory motif in the nucleotide-binding domain, stimulating constitutive activation of NF-κB [90]. Also, ER stress activates NOD2 in a ligand-independent way [91]. Otherwise, sequence variants of *NOD2* leucine-rich repeat (LRR) domain have been associated with susceptibility to Crohn’s disease [92]. BS usually presents early in life, before 5 years of age, with skin maculo or micropapular rash, granulomatous uveitis and symmetrical polyarthritis, with marked tenosynovitis and camptodactyly [93]. The presence of a particular *NOD2* genetic variant (c.2798 + 158C > Talone or associated with c.2023C > T-p.Arg675Trp) has been linked to the development of **Yao Syndrome or NOD2- associated AID (NAID)**, a multisystemic inflammatory disease presenting in adulthood [94].

#### 4.2.1. Ubiquitinopathies

Ubiquitination is a post-translational modification process, which is involved in the regulation of many cellular processes, including protein transcription, protein degradation, DNA-repair and endocytosis. It involves the linking of evolutionarily conserved 76-aa Ubiquitin (Ub) molecules to target proteins in the form of a monomer or polymers (Ub chains) through a step-by-step enzymatic pathway [95]. Seven lysine and the N-terminal methionine residues serve as linkers: for example, Lys63 (K63) Ub chains are involved in cell signalling and in DNA damage response, while linear (Met1) Ub chains regulate a wide range of immune signalling pathways [96]. The type of conjugation determines the fate of the modified protein and regulates protein localization, interactions, activities and degradation [95]. Furthermore, this process is dynamic and reversible, since Ub chains can be removed by a class of enzymes called deubiquitinases (DUBs) [97]. Both ubiquitination and deubiquitination are involved in the regulation of NF-κB pathway: ubiquitination stabilizes molecular complexes that promote TNF and IL-1β signalling and NF-κB activation, while deubiquitination is a negative regulator. The linear ubiquitin chain assembly complex (LUBAC) has been shown to maintain the stability of TNFR1, TLRs, IL-1 receptor (IL-1R) and other cytokine receptors. It is formed by heme-oxidized IRP2 ubiquitin ligase 1 L (HOIL-1), SHANK-associated RH domain-interacting protein (SHARPIN) and catalytic subunit HOIL-1L-interacting protein (HOIP). After stimulation with proinflammatory signals, LUBAC conjugates linear Ub chains to some target substrates (IKK, RIPk1, RIPK2, ASC), thus stimulating NF-κB and MAPK pathway activation. On the other hand, DUBs, like otulin and A20, reverse the effects of ubiquitination by hydrolysing linear (Met1) and Lys63 (K63) Ub portions, respectively, from conjugated protein [98]. Ubiquitination can also influence NLRP3 inflammasome activation [99]. Alterations at any level of this complex mechanism have been associated with the development of a type of autoinflammatory syndromes, called ubiquitinopathies. Patients with defects in the LUBAC components develop immunodeficiency, autoinflammation and muscular amylopectinosis with myopathy. AR loss-of-function *HOIP* mutations have been associated with splenomegaly, lymphangiectasia and B and T cell defects [100]. Patients with HOIL deficit have hepatosplenomegaly, lymphadenopathy and severe alteration of B cell function [101]. These pathogenic mutations have different effects according to the cell type: patients show compromised NF-κB responses in fibroblasts and B cell, causing recurrent bacterial infections, while their monocytes are hyperresponsive to IL-1β and produce high levels of proinflammatory cytokines IL-6 and Macrophage Inflammatory Protein 1α (MIP-1α) [98]. Recessively inherited loss-of-function mutations of the Family with Sequence Similarity 105, Member B (*FAM105B*) gene on chromosome 5 encoding for otulin have been associated with **Otulipenia/Otulin-related autoinflammatory syndrome (ORAS)**. Patients present early in life, usually within 3 months of age, recurrent prolonged fever, arthritis, diarrhoea, growth restriction and skin lesions, such as lipodystrophy, painful erythematous rash with skin nodules, pustules and panniculitis. Skin biopsy shows evidence for neutrophilic dermatitis, mixed type panniculitis and vasculitis of small and medium-sized blood vessels. In contrast to LUBAC deficiency, ORAS is not associated with immunodeficiency. A20 acts as a DUB, removing Ub chains from TNFR-associated factor 6 (TRAF6) and acting as a negative regulator of NF-κB.

#### 4.2.2. A20 Haploinsufficiency (HA20)

HA20 is due to AD missense mutations or small frameshift deletions of TNFα-induced protein 3 gene (*TNFAIP3*) on chromosome 6, mainly identified in the Japanese population [102]. The increasing diffusion of genome-wide analysis techniques has shown that common low-penetrance non-coding variants in *TNFAIP3* can be found in many autoimmune diseases including SLE [103], psoriasis [104], inflammatory bowel disease (IBD) [105], type 1 diabetes [106], and others. Most patients with HA20 present early in childhood with fever, uveitis, oral and/or genital ulcers, arthritis, skin involvement (dermal abscesses, folliculitis, papules) and ulcerative colitis with bloody diarrhoea, resembling Behcet’s disease [107]. Rarely, cerebral vasculitis and pulmonary embolism have been described. About 30% of patients show autoimmune features, including the presence of antinuclear (ANA) and anti-thyroid antibodies, while others presented with humoral immune deficiency, requiring immunoglobulin replacement therapy [108].

### 4.3. Deficiency of IL-1 Receptor Antagonist (DIRA)

DIRA is caused by AR loss-of-function mutations in *IL1RN* gene, encoding for the IL1 receptor antagonist protein (IL-1Ra) [109]. The disease presents with neonatal onset of skin pustulosis, multifocal osteomyelitis, periostitis of distal ribs and long bones and heterotopic bone formation [110]. The pathogenic mechanism is IL-1α and IL-1β hyperactivity, thus explaining the dramatic response to treatment with IL1R antagonist Anakinra [111].

### 4.4. Deficiency of IL-36 Receptor Antagonist (DITRA)

DITRA is an extremely rare AR disease, characterized by recurrent episodes of generalised sterile pustular rash, fever, neutrophilia and increased inflammatory markers. Oligoarthritis, glossitis and nail dystrophies have also been described. The attacks can present during the neonatal period or later in life and may be precipitated by stress, drugs or pregnancy. DITRA is caused by loss-of-function mutations of IL-36 receptor antagonist gene (*IL36RN*), causing decreased NF-κB inhibition and hyperinflammation, especially in keratinocytes [112].

## 5. Cytokine Signalling Disorders

### Adenosine Deaminase 2 (ADA2) Deficiency

Adenosine deaminase 2 deficiency (DADA2) was first described in 2014 in patients with a small- and medium-vessel vasculitis resembling polyarteritis nodosa (PAN) [113]. Unlike classic PAN, these subjects present with early onset disease and a broad spectrum of clinical manifestations, with vasculitis, haematological involvement and immunodeficiency. Intermittent fever, elevated inflammatory markers, skin vasculopathy (livedo reticularis, ulcers) and prominent neurological involvement, due to lacunar ischemic strokes and/or bleeding are the main clinical findings. GI tract, liver and kidney can also be involved. Moreover, vasculitis can be associated with hepatosplenomegaly, hypertension, haematological signs, from mild anaemia to pancytopenia, and immune dysregulation, in the form of hypogammaglobulinemia or lymphoproliferation [114,115]. The disease is caused by biallelic loss of function mutations of *ADA2* gene (also known as cat eye syndrome chromosome region candidate 1 *CERC1*) on chromosome 22 [113]. *ADA2* is expressed in myeloid cells. It plays an important role in monocyte proliferation and stimulates macrophage differentiation towards pro-inflammatory subset (M1) rather than an anti-inflammatory one (M2). Moreover, it stimulates proliferation of monocyte-activated CD4+ T cells [116]. A prominent IFN signature can also be found in DADA2 patients [117].

## 6. Type I Interferonopathies

Type I interferonopathies (IFNopathies) are a very recent group of inherited monogenic AIDs, characterized by a dysregulation of the type I IFN pathway, leading to constitutive upregulation of its activatory mechanisms or failure of negative regulatory systems [118,119]. According to the 2017 classification of the International Union of Immunological Societies (IUIS), 13 type I IFNopathies were identified [120]. However, this topic has recently been the object of an exponential interest and research in scientific literature and the number of genetic mutations, involved molecular mechanisms and corresponding clinical syndromes is rapidly expanding [118,119,121,122]. Nevertheless, it is possible to recognize five main molecular mechanisms leading to an altered regulation of IFN signal: (1) a cytosolic accumulation of endogenous nucleic acids due to loss of function mutations of genes encoding for DNA or RNA or DNA-RNA hybrid degradation enzymes; (2) reduction of the activation threshold or constitutive activation of intra-cytosolic nucleic acids sensors; (3) Gain-of-function mutations of positive IFN signalling regulators; (4) Loss-of-function mutations of negative IFN receptor signalling regulators; (5) proteasomal dysfunction, with unfolded protein response (UPR) activation and further downstream IFN pathway firing [119,121]. Despite clinical heterogeneity, some peculiar clinical aspects can be described, which are very different from other AIDs: recurrent “flu like” fever, early onset of skin vasculopathy with typical chilblains, livedo reticularis, panniculitis and later lipodystrophy, interstitial lung involvement with fibrosis or an early encephalopathic CNS involvement [119]. A precocious SLE habitus (SLE like-autoantibodies, cytopenia, glomerular renal involvement) represents another prominent feature of many of these pathologies [119,121,122].

### 6.1. Aicardi-Goutières Syndrome (AGS)

In 1984 Aicardi and Goutières described eight children suffering from severe early-onset encephalopathy of unknown origin, with CNS calcification and demyelination [123]. AGS was originally labelled as “pseudo-TORCH (toxoplasmosis, rubella, cytomegalovirus and herpes) syndrome”, identifying a group of serologically negative disorders that mimic congenital TORCH infections [118]. Later, in 2003, Crow et al. used the term “interferonopathy”, to underline the common pathological feature between AGS syndrome and viral congenital infections: an upregulation of interferon IFN-α activity [124]. The subsequent characterization of AGS-related gene mutations confirmed this hypothesis and AGS finally obtained a pathogenetic location, becoming the IFNopathies prototype [125,126,127]. AGS is defined as a progressive encephalopathy characterized by basal ganglia calcifications, chronic cerebrospinal fluid (CSF) lymphocytosis and elevated type I IFN levels in the CSF [119,127,128]. The estimated prevalence is 5–10/10000 children [129]. Seven gene mutations have been associated with AGS so far, leading to the identification of seven subgroups of the disease (AGS 1–7). AGS is mainly inherited in an AR manner and it results in mutations of genes encoding for intracellular nucleic acids degradation enzymes: 3′-5′ DNA exonuclease -*TREX1* (AGS1), ribonucleases as the ribonuclease H2 subunit B *RNASEH2B* (AGS2), ribonuclease H2 subunit C *RNASEH2C* (AGS3) and ribonuclease H2 subunit A *RNASEH2A* (AGS4), SAM and HD domain containing deoxynucleoside triphosphate triphosphohydrolase 1 *SAMHD1* (AGS5) and Adenosine deaminase acting on RNA 1 *ADAR1* (AGS6), causing inappropriate accumulation of endogenous nucleic acids [119,125,130,131,132,133,134,135,136]. AGS7 is a mild AD form, caused by mutations of genes encoding for the intracellular RNA sensor IFN-induced helicase C domain-containing protein 1 *IFIH1* (AGS7) [136].

Neurological symptoms appear within the first year of life, after a normal pregnancy and birth. Two forms of AGS have been described: in the early-onset form, psychomotor delay, feeding difficulties, irritability, episodes of aseptic febrile illness and microcephaly appear in the first month of life. Patients can develop important neurological sequelae, such as tetraparesis, trunk hypotonia and pyramidal or extrapyramidal signs (mainly dystonic postures and movements, spasticity and rigidity) [131,136]. “Startle reaction” is a peculiar dystonic manifestation, which appears even in response to minimal sensory stimuli [137]. Abnormal eye movements with nystagmus and glaucoma have also been described [133]. In late-onset AGS symptoms appear after months of normal development, as a progressive decline in head growth, slight spastic paraparesis and mild psychomotor retardation with slower progression and longer survival. Epileptic seizures are present in a variable percentage of patients (from 10–30% to 53–75%) and they need to be differentiated from dystonic movements [137,138]. Early stroke and cerebral aneurysms are significant clinical signs affecting the prognosis in patients with *SAMHD1* gene mutations [139]. The characteristic neuroimaging findings in AGS are intracranial calcifications (due to a microangiopathy with calcifications in vascular smooth muscle cell), leukodystrophy and brain atrophy, similar to those seen in congenital infections [119,134,136]. Deep white matter cysts, associated with *TREX1* mutations, and delayed myelination, associated with *RNASEH2B* mutations, have also been described [140]. Typically, the onset of neurological symptoms can occur in a hyperacute form, simulating a meningo-encephalitic infection or a metabolic disorder, followed by a subacute phase and then by clinical stabilization. Cases of radiological regression are even reported [141]. Extra-neurological involvement in AGS is present in 60% of patients, with often delayed onset compared to encephalopathy. Skin is the mainly affected organ (in 35% of cases): chilblain-like lesions, characterized by areas of inflammation and necrosis due to peripheral inflammatory vasculopathy, mainly localized in fingers and toes or in the auricles, mostly in the cold months. Hepatomegaly and a subacute hepatitis with slight increased transaminases and even a transient thrombocytopenia can be associated [119,130,131,132,133,134,136]. Moreover, a progressive contracting arthropathy was reported in children with *SAMHD1* gene mutations [142]. An autoimmune phenotype with type 1 diabetes mellitus, haemolytic anaemia, hypothyroidism or fluctuating SLE-like autoantibodies has been described in over 60% of cases [143].

### 6.2. Sting-Associated Vasculopathy with Onset in Infancy (SAVI)

SAVI is a monogenic early onset vasculitis due to a gain-of-function mutation in the transmembrane protein 173 (*TMEM173*) gene, encoding for STING, which induces IFN pathway activation by foreign DNA [144]. Skin and lungs are the most affected organs. Telangiectatic lesions on nose and cheek, violaceous atrophic plaques and nodules on hands, painful ulcerative lesions evolving eschars and even digital amputation, ear cartilage reabsorption, perforation of the nasal septum, periungual erythema and onychodystrophy are the main coetaneous manifestations. Cold exposure may trigger cutaneous flares. Respiratory tract involvement is characterized by interstitial lung disease with progressive fibrosis and hilar or paratracheal persistent lymphadenopathy. A progressive hypoxemic respiratory insufficiency, with a restrictive pattern in spirometry, needing for a chronic oxygen supplementation can be identified from late childhood to adolescence [145]. High title ANA and anti-neutrophil cytoplasmic antibodies (cANCA) were discovered in some patients, resulting in a difficult differential diagnosis with childhood granulomatosis and polyangiitis [146]. Fever spikes, chronic anaemia and growth failure are other clinical non-specific features of SAVI [144,145]. Phenotype mimicking the rheumatoid factor (RF) positive polyarticular juvenile idiopathic arthritis (JIA) associated with interstitial lung disease or inflammatory myositis have also recently been reported [147].

### 6.3. Monogenic Systemic Lupus Erythematosus

The presence of increased IFNα levels in SLE patients’ sera, that also correlated with disease activity, is largely known, suggesting a pathogenetic role of type I IFN in SLE clinical manifestations [148,149,150,151,152,153,154,155]. Recently, childhood-onset SLE has been linked to single gene mutations, defining monogenic or Mendelian SLE. At least three classes of gene mutations have been described [156,157]. Firstly, complement factors defects (above all C1q and C4 factors), leading to a defective opsonization of apoptotic self-bodies and, therefore, to the cellular clearance and the “efferocytosis”, represents the most frequent form. In particular, genetic deficiency of the early component of the classical pathway *C1q/r/s* strongly predisposes to SLE, with a penetrance of up to 90%. The clinical phenotype is represented by an early onset of SLE with frequent early renal glomerular involvement and photosensitive skin rash associated with an increased susceptibility to pyogenic infections, eventually even life-threatening infections such as meningitis [119,156]. Secondly, the role of endonuclease gene defects in the development of Mendelian SLE was recently demonstrated [156,157,158,159]. DNAse1, mainly DNAse1-L3, displays a prominent role in digesting genomic DNA circulating “microparticles” derived from apoptotic cells, leading to the accumulation of extracellular nucleic acids which will be recognized by DNA/RNA sensors, thus promoting type I interferon production [160,161,162]. Clinical and laboratory aspects of DNAse1-L3 deficiency include an early onset nephritis with high title multiple autoantibodies (ANA, anti-dsDNA and ANCA) [156,160]. A form of hypocomplementemic urticarial vasculitis, with dermal vasculitis, arthritis and glomerulonephritis was also described [163]. Furthermore, a type I IFN-mediated autoinflammatory phenotype due to DNAse2 deficiency, (a lysosomal endonuclease that degrades cytosolic erythroblast nuclei DNA) was also more recently described. Biallelic mutations in *DNAse2* gene were associated with a typical clinical picture, characterized by neonatal onset hepatosplenomegaly, cholestatic hepatitis and pancytopenia with a later membrano-proliferative glomerulonephritis, deforming arthropathy, foot vasculitis and constant presence of anti-DNA antibodies [164]. Finally, the third group includes genes encoding for enzymes involved in the endogenous nucleic acid degradation and directly involved in the IFN signal. Mostly, AD mutations of *TREX1* (DNA 3′—repair exonuclease) are related to the early-onset **familial chilblain lupus (FCL)**. It is a rare form of cutaneous SLE characterized by cold-induced severe vasculitic ulcerative lesions of finger of hands, feet and ears [165,166,167,168]. In addition, mutations in *TREX1* with AD inheritance were related to **retinal vasculopathy with cerebral leukodystrophy (RVCL)**. RVCL is characterized by middle-age onset of progressive visual loss due to retinal vasculopathy (telangiectasias, microaneurysms, retinal macular vascular obliteration) and neurologic manifestations including hemiparesis, facial paralysis, aphasia and hemianopsia up to psychiatric disorders [169]. Furthermore, a homozygous variant of this gene was identified in patients with an early-onset cerebral SLE [170]. Also, mutations in *SAMHD1* have been reported in patients affected by FCL, with and without vascular CNS involvement [171].

### 6.4. Proteasome Associated Autoinflammatory Syndromes (PRAAS)

Inherited or de novo loss-of-function mutations in genes encoding for proteasome subunits of the 20S core particle (as *PSMB8, PSMB9, PSMB7, PSMA3, PSMB10*), or proteasome chaperone factors (as Proteasome Maturation Protein or *POMP* and proteasome assembly chaperone 2 *PSMG2*) are the genetic substrates of a heterogeneous class of IFNopathies, named PRAAS [172,173,174,175,176,177,178]. The main diseases that can be included in this group are the **joint contractures, muscle atrophy, microcytic anaemia and panniculitis-induced lipodystrophy syndrome (JMP)**, the **Nakajo-Nishimura syndrome** and **chronic atypical neutrophilic dermatosis with lipodystrophy and elevated temperature syndrome (CANDLE)** [119]. The common PRAAS underlying pathogenetic mechanism is the failure of proteasome complex formation, leading to the intracellular accumulation of ubiquitinated proteins and compromises the ER-associated proteins degradation, resulting in an accumulation of misfolded ER proteins in the lumen. The subsequent ER stress is capable of activating UPR pathway, that allows NF-κB activation and IRF3 factors transcription, starting an IFN-depending inflammation [175]. Typical CANDLE’s clinical features are the presence of pernio-like purplish nodular lesions (neutrophilic dermatosis), panniculitis with progressive lipodystrophy and recurrent hyperpyrexia. Eyelid and digital swelling are frequently found in the acute phases. An early metabolic syndrome, with systemic hypertension and dyslipidaemia, occurs in 40–80% of patients [173,174,175]. Muscle atrophy, joint contractures with extremities deformity, hepatosplenomegaly and hypochromic or haemolytic anaemia were also reported. Very recently, a peculiar hypo-hyperinflammatory phenotype has been described in two unrelated individuals: the **POMP-related autoinflammation and immune dysregulation disease (PRAID)**, characterized by early-onset combined immunodeficiency, inflammatory neutrophilic dermatosis and multiple autoimmunity. Genetic analysis revealed heterozygous frameshift mutations of the exonic *POMP* chaperone, which by escaping nonsense-mediated mRNA decay (NMD), results in a truncated protein that perturbs proteasome assembly by a dominant-negative mechanism [178].

### 6.5. COPA Syndrome

A familial form of high title RF positive juvenile “idiopathic” arthritis and associated haemorrhagic interstitial lung disease are the pivotal clinical manifestations of the very recently described COPA syndrome [179]. Chronic wheezing and typical lung cysts chest pain can also occur in these patients, with gradual decline in lung function. Moreover, alveolar haemorrhaging is the most important potentially life-threatening complication with acute respiratory failure and anaemia. Genetic analysis of these subjects allowed to detect heterozygous mutations in the gene coding for the coatomer complex subunit alpha (*COPa*), which plays a role in the intra-cytosolic proteins’ retro-transport between Golgi apparatus and ER. COPA clinical expression and penetrance appear to be very heterogeneous [180]. The presence of common clinical and pathogenetic features to SAVI syndrome and the finding of the type I IFN pathway activation in peripheral blood of COPA syndrome patients allowed the identification of a role of IFN signal dysregulation in the pathogenesis of this disease [179,180]. Moreover, according to current evidence, Type I IFN is triggered by intracellular ER stress, activating pathways which overlap with those previously described for the PRAAS [180]. Very recently, another interesting underlying mechanism has been described: loss of COPA function causes a failure of Golgi-to-ER STING retrieval, with accumulation of ER-resident STING at the Golgi and further constitutive IFN next signal propagation [180,181].

## 7. AIDs: Current Diagnostic Tools

Given the extreme complexity and variability of this group of syndromes, the diagnosis of AIDs still represents a challenge for paediatricians. The first step is clinical suspicion, guided by the awareness and subsequent identification of the hallmarks of disease. Inflammasomopathies are characterized by recurrent episodes of fever and inflammatory markers elevation, with associated lymphadenopathy, hepatosplenomegaly, chronic arthritis, serositis, rash, GI symptoms and/or neurological involvement. More rarely, vasculopathy and haematological alterations can be associated. These typical signs are associated with elevated inflammatory markers (CRP, erythrocyte sedimentation rate ESR, SAA) during attacks. Amyloidosis is a frequent complication. General practitioners should consider the diagnosis of IL-1-related diseases in children with periodic recurrence of inflammatory symptoms, in the absence of other recognizable causes, such as chronic infections and malignancies. The presence of similar cases in the family is another important diagnostic clue.

The type I IFN clinical autoinflammatory phenotype is very different from the IL1- mediated one (Table 5). An early necrotizing vasculitis, a non-infectious interstitial lung disease in the context of an inflammatory clinical picture, a panniculitis with or without associated lipodystrophy and early thrombotic events are peculiar anamnestic elements, that should lead to the suspicion of an IFNopathy [119,121]. A differential diagnosis with these pathologies has to be considered by the paediatric rheumatologist in case of JIA-like polyarthritis, but refractory to conventional treatments and occurring in several members of the same family or in case of very early-onset childhood SLE, with prominent skin and kidney involvement [119,122]. Moreover, in contrast to patients with inflammasome-mediated AIDs, patients with interferonopathies could present with elevated autoantibody titres, including ANA, extractable nuclear antigen (ENA), c-ANCA, anti-thyroid antibodies, lupus anticoagulant (LAC), anti-cardiolipin and beta-2-glycoprotein I (β2GPI) antibodies, which is consistent with the prominent role of Type-1 IFN in driving autoantibody formation. The presence of high fluctuating titres of such antibodies should represent another suspicious laboratory element [119,172]. The most important clinical and laboratory hallmarks of AIDs and IFNopathies are summarized in Table 5.

The second step for diagnosis is genetic analysis. Sozeri et al. recently proved that clinical diagnostic criteria may not always be sufficient to establish the correct diagnosis and that screening of a restricted number of candidate genes or exons by Sanger sequencing may be the cause of negative genetic results. Therefore, NGS panel may improve genetic diagnosis, allow the understanding of the pathogenetic mechanism and reduce diagnostic delay [182]. Furthermore, in case of suspicion, screening for IFN signalling should be another diagnostic step. Type I IFN protein dosage is not available in routine clinical practice because of its very low circulating levels. However, type I IFN pathway upregulation can be investigated with the increased expression of a subset of six ISGs (IFI27, IFI44L, IFIT1, ISG15, RSAD2 and SIGLEC1) by quantitative polymerase chain reaction (PCR) assays with whole blood samples, the so-called “IFN signature” [119,122,183]. The IFN signature seems to be very sensitive to differentiate monogenic type I interferonopathies from inflammasomopathies and also polygenic pathologies predominantly IFN mediated (such as SLE) by pathologies whose inflammatory component is different from IFN, such as non-systemic JIA. Starting from the concept of “IFN signature”, a different “IFN score” was developed with a high sensitivity for AGS and other IFNopathies [183]. However, standardization between different centres could be difficult [122]. Therefore, new “IFN scores” have been proposed for autoimmune diseases such as SLE or antiphospholipid antibody syndrome (ALS) using NanoString technology and new standardization methods are being studied to make the reporting homogeneous in research laboratories [184,185,186]. Since IFNopathies are hereditary genetic diseases, the conclusive diagnostic step is genetic sequencing. Currently, not only first level genetic tests searchingfor known mutations using specific gene panels, but also second and third level genetic testing and NGS (exome or whole genome sequencing) are available [119,121,122].

## 8. Current Therapeutic Approaches in AIDs

### 8.1. Inflammasomopathies

Recent advances in genetic analysis techniques have allowed deeper understanding of the molecular mechanisms of AIDs, paving the way for targeted treatment. Until the 90’s, management of patients with recurrent inflammatory symptoms was based on the use of traditional drugs, such as glucocorticoids and non-steroidal anti-inflammatory drugs (NSAIDs), often with limited efficacy on acute symptoms. NSAIDs can be used in monogenic AIDs as symptomatic treatment for pain relief during inflammatory attacks, alone or with baseline therapy, with complete response in a minority of patients [187]. An exception is represented by **protracted febrile myalgia**, a condition associated with FMF and characterized by prolonged limb muscular pain, marked systemic inflammatory response and no signs of rhabdomyolysis. In these patients, NSAIDSs seem to be effective [188], as also intravenous or oral administration of high dose glucocorticoids [189]. Short-term glucocorticoids are largely used in FMF, TRAPS, CAPS and MKD on demand, but their use has not proven to reduce either the intensity and frequency of the attacks, or the risk of amyloidosis [187,190]. Colchicine is an alkaloid extracted from plants, with anti-inflammatory properties, due to the effect on microtubules organization. It inhibits leukocyte chemotaxis, neutrophil binding to vascular endothelium, TNF-α production by macrophages and TNF-α receptors expression on endothelial cells [191]. It also acts on phospholipase A2 activity, lysosomal mechanisms and phagocytosis [192]. Colchicine represents the first line of treatment in patients with FMF, with consistent reduction on attack frequency and effective prevention of secondary amyloidosis. However, 2% of patients do not tolerate or are not responsive to this drug [193]. Colchicine has not demonstrated effectiveness in the treatment of MKD and CAPS, while it can show some results in TRAPS [187]. Recently, Vitale et al. retrospectively enrolled 24 patients with TRAPS treated with colchicine in monotherapy. They reported a complete response in 12.5% of cases and a partial response in 58.3%, with no differences between age of onset and underlying genetic variant, concluding that colchicine monotherapy could be useful in a low percentage of TRAPS patients and attempted in patients with milder phenotypes and at a lower risk of developing reactive amyloidosis [194]. The discovery of the increased production of IL-1β as the main pathogenetic mechanism in many inflammasomopathies led to the introduction of anti-IL1 agents and other biologic agents in the management of AIDs. IL-1 blockade has become the most specific and effective treatment for inflammasome-mediated AIDs, such as FMF, TRAPS, MKD and CAPS, as first line therapy or when previous conventional treatments are not effective [195]. Their use results in sustained reduction of disease activity, but chronic treatment is often required. The increasing scientific interest in this field stimulated the diffusion of randomized controlled clinical trials exploring the safety and efficacy of these drugs [196]. The three available anti-IL1 biologic agents are Anakinra, Canakinumab and Rilonacept. Anakinra is a recombinant non glycosylated form of IL-1 receptor antagonist (rhIL-1Ra), which binds to IL-1 receptor type I (IL-1RI), acting as competitive inhibitor with IL-1α and IL-1β. It was approved for all types of CAPS by the U.S. Food and Drug Administration (FDA), following a long-term open-label study on 18 CINCA/NOMID patients, showing lasting clinical and laboratory remission in all patients, in addition to improvement in cochlear and leptomeningeal lesions at magnetic resonance imaging (MRI) [197]. Later, also European Medicine Agency (EMA) approved this drug in adults, adolescents and children aged 8 months or older with CAPS, basing its decision on several open-label and perspective studies and case series [198,199]. Kuemmerle-Deschner J.B. et al. [200] performed a single-center observational study on 12 patients with severe MWS (five children and seven adults) receiving Anakinra for a median of 11 months. Patients showed organ manifestations improvement, together with a parallel decrease in S100A12 serum levels. Treatment was well tolerated, without serious adverse events. A prospective, open-label single center clinical cohort study investigated the efficacy and safety of Anakinra treatment for up to 5 years in 43 patients with CAPS. The drug was safe and tolerated both in paediatric and adult patients and the most frequent adverse events were headache, arthralgia and injection site reactions; infections were described in 25% of patients but did not require permanent discontinuation of therapy [201]. Similarly, many studies showed the efficacy of anakinra in the treatment of colchicine resistant FMF [202], TRAPS [203] and MKD [204]. Canakinumab is a humanized monoclonal anti-IL-1β antibody. It was approved by FDA and EMA in 2009 for adults and children older than 2 years with CAPS. Some authors [205] reported the results of a multicenter study conducted on 109 canakinumab-naïve paediatric and adult patients with CAPS and 57 patients previously treated with canakinumab. Complete response was achieved in 78% of naive patients. The drug was administered for up to 2 years and 90.4% of patients experienced adverse events, mainly mild, such as headache, rhinitis, arthralgia, diarrhoea and upper respiratory tract infections. Patients receiving vaccination (15%) showed normal immune response. Later, in 2016, Canakinumab received FDA and EMA approval for the therapy of colchicine resistant FMF, MKD and TRAPS, based on the results of the phase 3 Canakinumab Pivotal Umbrella Study in Three Hereditary Periodic Fevers (CLUSTER) trial [206]. Many studies investigated and proved the efficacy of canakinumab in these diseases [207,208,209]. Rilonacept is a fusion glycoprotein consisting of the Fc portion of human IgG1 and the human IL-1R domain. In 2008, a placebo-controlled phase III trial reported symptoms improvement, SAA levels reduction and a favourable tolerability profile in adult patients with CAPS treated with weekly rilonacept. Thus, this drug was approved by FDA for the treatment of FCAS and MWS in patients older than 12 years [210]. Hereafter, Hoffman H.L. et al. showed that rilonacept reduces the frequency and duration of attacks in adult and paediatric patients with CAPS, in the absence of severe adverse effects (injection site reactions, headache and upper respiratory and urinary tract infections have been described) [211]. Anti-TNF agents, mainly etanercept, infliximab and adalimumab have been extensively used in many AIDs. Etanercept has been proved to prevent or reduce the intensity of attacks and the dose of glucocorticoids in patients with TRAPS, but it is often discontinued due to loss of efficacy [212]. In a multicenter retrospective international study 41 patients with pathogenic TRAPS mutations and 6 with the R92Q were treated with etanercept. In the first group 50% of subjects achieved a complete clinical response, but 65% of patients had to discontinue treatment due to loss of efficacy; in the second group, only 25% had a complete response. Anakinra, instead, showed greater and lasting efficacy [213]. On the other hand, infliximab and adalimumab have been associated with severe paradoxical reactions and therefore they are not recommended in TRAPS [214]. In MKD patients, anti-TNF therapy can improve frequency and intensity of attacks, but it is recommended as second-line option in case of IL1 blockade is ineffective or untollerated [83]. Finally, tocilizumab, a humanized monoclonal anti-IL6 receptor antibody, has not been approved jet for the treatment of AIDs. However, some case reports documented a good control of disease activity and of secondary amyloidosis, in patients with colchicine resistant FMF [215,216]. This drug has also proved its effectiveness over proteinuria in FMF related amyloidosis [217].

### 8.2. Other Monogenic AIDs

As regards the other monogenic AIDs, clear therapeutic indications are lacking and pharmacological management is based on clinical experience and anecdotal case series. For example, many therapeutic options have been employed in patients with BS, according to the severity of organ involvement: the most commonly used drugs are corticosteroids, methotrexate, mycophenolate mofetil, adalimumab, infliximab and canakinumab, but anti-TNF seem to be the best therapeutic option [218]. Blau-associated uveitis has been successfully treated with steroids and anti-IL1 [219]. In children with ORAS, a good disease activity control is achieved with TNF inhibitors, while anti-IL1 have shown partial effects [220,221]. Finally, HA20 patients have high serum levels of proinflammatory cytokines produced by myeloid cells (IL-1, TNF, IL-6, IL-18) and T cells (IL-9, IL-17, and IFNγ). Indeed, therapies with TNF and IL-1 inhibitors are often employed [222]. Furthermore, the presence of a type I IFN signature predicts a good effectiveness of anti-JAK therapy in patients with poor response to anti-cytokines [223]. About DIRA, the pathogenic mechanism is IL-1α and IL-1β hyperactivity, thus explaining the dramatic response to treatment with IL1R antagonist Anakinra [111]. Optimal treatment for DITRA has not been defined yet, but good clinical responses have been described with anti-IL1 Anakinra [224], anti-TNF infliximab [225], anti-IL17 secukinumab [226] and anti-IL12/23 ustekinumab [227].

### 8.3. IFNopathies

Conversely, Type I IFNopathies are often resistant to conventional biologic treatments [119,121,122]. The only exception is the possible efficacy of anti-IL6 drugs for neuropathy in patients with *SAMDH1* mutation [228]. The corticosteroids response is often partial, requiring high dosages, being also often associated with subsequent steroid-dependence [119,121,229]. Some antimalarial drugs, such as hydroxychloroquine, have been shown to modulate the initial phases of the IFN cascade but their role in IFNopathies can be identified only in association with other drugs [230]. Indeed, the most promising therapeutic strategy is represented by JAK inhibitors (baricitinib and ruxolitinib selective JAK1/JAK2 inhibitors and tofacitinib JAK1/JAK3 blocker), acting directly on the JAK-STAT signalling pathway. This hypothesis, which had already been confirmed by in vitro studies and sporadic case reports, has recently been demonstrated [231,232,233,234]. Sanchez et al. enrolled 18 children (10 with CANDLE, four with SAVI and four with genetically undefined pathology) for treatment with baricitinib. After a mean of 3 years of therapy, 67% of patients have shown a statistically significant improvement in clinical items and 71% of them a reduction in the need for glucocorticoids. This effect, already present at the beginning of treatment, is increased until optimal doses are reached, remaining stable in the 90 days before the final visit. Additionally, height and bone mineral density Z-scores significantly improved, and their IFN biomarkers decreased [234]. However, JAK inhibitors may only partially control disease activity in patients with PRAAS. Martinez et al. recently reported the successful use of HSCT in two individuals with POMP deficiency, suggesting that the clinical and immunological features of PRAID could be derived from a proteasome defect in hematopoietic cells. Therefore, HSCT should be considered in patients with life-threatening disease [235].

Antiretroviral therapy (RT therapy) used for HIV infection is another interesting option under investigation. It is known that more than half of the human genome is formed by retrostrasposomes, capable of moving from within the genome by a reverse transcription of an RNA intermediate (complementary DNA cDNA). In vitro and murine studies have shown that human cDNA may be substrate of TREX, SAMHD1 and ADAR enzymes. Besides, cDNA accumulation could be an important cause of activation of IFN pathway. Moreover, *TREX1* knockout mice or reprogrammed neurons have a reduction in IFN clinical inflammation by treatment with a combination of RT inhibitor [236,237]. Recently, the results of a single-center, open-label pilot study involving 11 patients with AGS treated with abacavir, lamivudine and zidovudine for 12 months have been published, demonstrating a reduction in IFN signature and IFN activity in the CSF. Future studies with clinical outcomes to confirm these data are required [238]. Finally, the new monoclonal antibodies targeting IFN-α (sifalimumab) and IFNAR (anifrolumab), whose efficacy has recently been demonstrated in SLE patients, represent another promising option for IFNopathy patients [119,122,239,240].

## 9. From Present Knowledge to Future Advances

### 9.1. What Do We Learn from AIDs?

The ever-increasing identification of the detailed molecular mechanisms underlying flogosis in monogenic AIDs is making a fundamental contribution to delineate the pathogenetic patterns of polygenic AIDs, bringing to light innovative therapeutic targets. Evidence of inflammasome activation have been found in many polygenic AIDs. In fact, the activation of inflammasomes leads to T helper 1 (Th1) and Th17 differentiation, shaping the adaptive immune response and favouring the development of autoimmune or chronic inflammatory pathways [10]. For example, the involvement of NLRP3 inflammasome dysregulation in Kawasaki disease (KD) has raised great interest [241], like its involvement in juvenile SLE (JSLE) [242], inflammatory bowel disease (IBD) [243] and systemic juvenile idiopathic arthritis (sJIA) [244]. Gene expression studies in sJIA reported IL-6 and TLR/IL-1R pathway hyperactivation, in addition to upregulation of AIM2 and NLRC4 in neutrophils and persistently elevated IL-18 levels despite clinically inactive disease [245]. Yang et al. reported that the variant rs4353135 G allele carrier of *NLRP3* gene conferred increased risk for oligoarticular and polyarticular JIA in a Taiwanese population, and that these patients had increased macrophage IL-1β production and Th17 response. Interestingly, they responded well to TNF therapeutic inhibition [246]. Similarly, other inflammasome genetic variants, like *MEFV* rs224204 and *NLRP3* rs3806265, can be associated with susceptibility to psoriatic JIA (pJIA) [247]. Furthermore, sJIA patients with higher levels of IL-18 are more likely to develop MAS, so that IL-18 levels could have a prognostic value and guide future therapeutic options, even in patients well controlled by other biologics, including anti-IL6 and anti-IL1 [248]. Even juvenile dermatomyositis (JDM) has been associated with TNF-α and IL-1 cytokines genetic polymorphisms, and TNF-α levels have been shown to correlate with disease activity [249]. Moreover, the characterization of the close interplay between innate and adaptive immune systems has dissolved the dividing line between autoinflammatory and autoimmune disease. The definition of the IFN pathway and the discovering of the pronounced autoimmune component of IFNopathies have played a prominent role in this acquisition. Production of Type I IFN by specialized plasmacytoid dendritic cell in response to various stimuli, such as neutrophilic NETosis, seems to be the key initiating event shared by most of these autoimmune diseases [250]. Several studies demonstrated an “IFN signature” in the early stages and event before the development of childhood onset SLE, Sjogren’s syndrome, inflammatory myositis, type 1 diabetes mellitus, autoimmune pancreatitis and thyroiditis and in IgG4- related disorders [250,251,252,253,254]. This finding offers a very promising target for therapy and even prevention of such autoimmune diseases. Moreover, the integration between IFN signature analysis and other laboratory indices, such as complement levels, seems to help to stratify paediatric SLE patients into two groups, in which the autoimmune or autoinflammatory component of the disease are prevalent, with different response to treatment [255].

### 9.2. New Insights and Perspectives

At least 40–60% of patients with typical phenotypes for AIDs fail to receive a specific diagnosis, leading to the definition of undifferentiated systemic autoinflammatory diseases (USAIDs). Besides, according to recent reports, the timeline from onset of symptoms to the diagnosis takes up to 7.3 years [6]. Nevertheless, these data could be modified very shortly, thanks to the exponential advent of NGS technologies. Indeed, very recently, De Jesus et al. applied a sequential algorithm characterized by the combination of a clinical phenotyping, a standardized type-I IFN response gene score (IRG-S), a targeted cytokine profile and the genetic evaluation by NGS to 66 USAID patients. Through this process, only seven patients remained unclassified and three new IFN-autoinflammatory diseases were identified: the **IL-18–associated pulmonary alveolar proteinosis and MAS syndrome (IL-18PAP-MAS)**, the **NEMO deleted exon 5–autoinflammatory syndrome (NEMO-NDAS)** and the **SAMD9L-associated autoinflammatory disease (SAMD9L-SAAD)**. Moreover, patients characterized by these new mutations had a IRGs profile that suggests a prominent NF-κB activation, very different from typical IFNopathies [256]. This finding paves the way for new interactions between inflammatory signalling pathways in AIDs and for new interesting therapeutic targets. The further exploration of intracellular pathways currently not explicitly involved in monogenic AIDs, like NOCHT signalling above all, could represent another research starting point [257]. Furthermore, the study of epigenetics is opening up new possible scenarios also in monogenic AIDs. Currently, epigenetic changes, as DNA methylation, microRNA (miRNA) expression, or histone modifications can be efficiently analysed with microarray and NGS approaches. Altered methylation levels of the CpG island, in *MEFV* gene in peripheral leukocytes, seems to explain the clinical expressiveness of a group of FMF patients affected by a single *MEFV* canonical mutation [258]. Also, the role of miRNAs, which are small single stranded RNA molecules that regulate gene expression by base-pair binding to mRNA, is an option under study [6]. A differential expression of miRNA in the acute and remission phases in FMF and TRAPS patients could be shown. For example, it has been shown that NLRP3 inflammasome activity is negatively controlled by miR-223 and its expression is associated with NLRP3-AID [259,260]. Additionally, proteomics and metabolomics can be used to identify molecular-pathological layers in AIDs in the near future by cooperative researches [6]. Based on the NIH, 147 clinical trials for “autoinflammatory disease” are currently active or recently concluded, allowing us to hope for considerable acquisitions in AIDs for the next few years [261].

## 10. Conclusions

AIDs are extremely heterogeneous syndromes, with a variable clinical phenotype, which is also influenced by genetic background and unknown epigenetic mechanisms. Recognition of the hallmarks of disease and exclusion of chronic infections and malignancies are the first steps of diagnosis, which is mainly guided by clinical suspicion. Therefore, general paediatricians must be aware of this group of diseases, in order to guide further examinations and refer the patient to a specialist rheumatologist. On the other hand, increasing research is focusing on the molecular pathogenic mechanisms. Such detailed flogosis signalling characterizations are the cornerstone of the current trend in paediatric rheumatology to reorganize from an “organ-based disease classification” of diseases to an “inflammatory pattern-disease classification”, in order to identify immediately the potential tailored therapeutic targets.

## Figures and Tables

**Table 1:** Monogenic AIDs overview.

INFLAMMASOMOPATHIES						
DISEASE	GENE	LOCUS	PROTEIN	PATHOGENESIS	CLINICAL HALLMARKS	INHERITANCE
**FMF**	*MEFV* (NM_000243.2)	16p13.3	Pyrin	Gain-of-function mutations causing poor affinity to regulatory proteins (PKN1, PKN2, 14-3-3) -> constitutive activation of pyrin inflammasome	Fever (12–72 h), serositis (abdominal pain, chest pain), non-erosive acute arthritis of large joints, erysipela-like lower extremity rash	AR/AD
**PAAND**	*MEFV* (S242R mutation)	16p13.3	Pyrin	Loss of pyrin inhibition by 14-3-3 protein	Fever, neutrophilic dermatosis, acne, pyoderma gangrenosum, cutaneous abscesses	AD
**MKD**	*MVK* (NM_000431.4)	12q24.11	Mevalonate kinase	↓ prenylation of proteins, necessary for Rho-A activation and PI3K-mediated inhibition of pyrin inflammasome	Early-onset (<1 year), fever (3–7 days), GI symptoms, arthromyalgia or arthritis, maculo-papular or urticarial rash, aphthous stomatitis, hepatosplenomegaly, cervical adenopathy	AR
**PAPA**	*PSTPIP1* (NM_003978.5)	15q24.3	CD2-binding protein 1 (CD2-BP1)	Gain-of-function mutations of CD2-BP1, which interacts with pyrin and enhances pyrin inflammasome activation	Pyoderma gangrenosum, arthritis, acne	AD
**PFIT**	*WDR1* (NM_017491.5)	4p16.1	WD40 repeat protein 1	Hypomorphic mutation, actin accumulation, pyrin inflammasome dysregulation, ↑ IL18	fever (up to 7 days), mucosal ulcerations, thrombocytopenia, infections	AR
**CAPS**	*NLRP3* (NM_004895.4)	1q44	Cryopyrin	Gain-of-function mutations of cryopyrin -> ↑caspase-1 activity -> ↑ active IL-1β	FCAS: cold-triggered episodes of fever, urticaria, conjunctivitis	AD
					MWS: cold-urticaria, sensorineural hearing loss	
					CINCA: neonatal onset, urticaria, chronic aseptic meningitis, deforming arthropathy, facial dysmorphia	
**FCAS4**	*NLRC4* (NM_001199138.2)	2p22.3	NLRC4	Gain-of-function mutations, NLRC4 inflammasome hyperactivation, ↑ IL-1β and IL-18	neonatal-onset, cold-induced urticaria, arthralgia, fever	AD
**AIFEC**	*NLRC4* (NM_001199138.2)	2p22.3	NLRC4	NLRC4 inflammasome hyperactivation	Early onset enterocolitis, recurrent MAS	AD
**FCAS2**	*NLRP12* (NM_144687.3)	19q13.42	Monarch 1	↓ constitutive NF-κB inhibition, ↑ ROS production	cold-induced urticaria, arthralgia, fever	AD
**DISORDERS OF TUMOR NECROSIS FACTOR (TNF)/NF-κB ACTIVITY**						
**DISEASE**	**GENE**	**LOCUS**	**PROTEIN**	**PATHOGENESIS**	**CLINICAL HALLMARKS**	**INHERITANCE**
**TRAPS**	*TNFRSF1A* (NM_001065.4)	12p13.31	TNF receptor superfamily member 1A	Altered intracellular TNFR trafficking, ER stress, ↑ ROS, ↑ NLRP3 inflammasome activation	Fever (>7–14 days), periorbital oedema, conjunctivitis, pseudo-cellulitis rash, abdominal pain, migrating myalgia, arthralgia, chest pain, lymphadenopathy	AD
**BS**	*NOD2* (NM_001370466.1)	3p21	NOD2/CARD15	Gain-of-function mutations, ↓ auto-inhibition of NF-κB pathway	< 5 years of age, rash, granulomatous uveitis, symmetrical polyarthritis	AD
**LUBAC deficiency**	*HOIL1* (NM_031229.4) *HOIP* (NM_017999.5)	14q12	HOIL1 HOIP	Defective deubiquitination, constitutive hyperactivation of NF-κB	fever, immunodeficiency, hepatosplenomegaly, amylopectin-like deposits in muscles	AD AR
**ORAS**	*FAM105B* (NM_138348.6)	5p15.2	Otulin	Loss-of-function mutations, defective deubiquitination, constitutive hyperactivation of NF-κB	Onset < 3 months, fever, diarrhoea, arthritis, lipodystrophy, panniculitis, growth restriction	AR
**HA20**	*TNFAIP3* (NM_001270508.2)	6q23.3	A20	Loss-of-function mutations, defective deubiquitination, constitutive hyperactivation of NF-κB	Fever, oral, GI and genital ulcerations, arthritis, uveitis (dd Behcet’s disease)	AD
**OTHER MONOGENIC AIDs**						
**DISEASE**	**GENE**	**LOCUS**	**PROTEIN**	**PATHOGENESIS**	**CLINICAL HALLMARKS**	**INHERITANCE**
**DADA2**	*CERC1* (NM_001282225.2)	22q11.1	ADA2	Macrophage differentiation towards M1 pro-inflammatory activity, IFN signature	Fever, vasculitis (livedo reticularis, ulcers), stroke, cytopenias, hypogammaglobulinemia	AR
**DIRA**	*IL1RN* (NM_173841.2)	2q14.1	IL1Ra	↓ IL-1α and IL-1β inhibition	Pustular rash, multifocal osteomyelitis, periostitis	AR
**DITRA**	*IL36RN* (NM_173170.1)	2q14.1	IL36Ra	↓ NF-κB inhibition, hyperinflammation in keratinocytes	Pustular psoriasis, fever, neutrophilia	AR
**TYPE I INTERFERONOPATHIES**						
**DISEASE**	**GENE**	**LOCUS**	**PROTEIN**	**PATHOGENESIS**	**CLINICAL HALLMARKS**	**INHERITANCE**
**AGS 1**	*TREX-1* (NM_130384.3)	3p21.31	3′-5′ DNA exonuclease	Accumulation of nucleic acids	Basal ganglia calcifications, delayed psycho-motor development, epilepsy (classic AGS)	AR /AD
**AGS2**	*RNASEH2B* (NM_001142279.2)	13q14.3	RNAse H2 complex. RNA-DNA hybrids degradation		Classic AGS	AR
**AGS3**	*RNASEH2C* (NM_182710.3)	11.q.13.1			Classic ASG	AR
**AGS4**	*RNASEH2A* (NM_006397.3)	19p13.13			ASG + dysmorfic features	AR
**AGS5**	*SAMHD1* (NM_080628.3)	20q11.23	SAM domain and HD domain-containing protein 1 (restricts the availability of deoxynucleotides)		Severe neurological involvement with early stroke, arthropathy	AR
**AGS6**	*ADAR* (NM_001111.5)	1q21.3	adenosine deaminase, RNA-specific		ASG, bilateral striatal necrosis	AR/AD
**AGS7**	*IFIH1* (NM_022168.4)	2q24.3	Interferon Induced with Helicase C Domain 1 (Cytosolic receptor for dsRNA)		Mild AGS	AD
**C1QA/B/C**	C1q (NM_015991.4)	1p36.12	Complement—Classic pathway	Complement pathway dysregulation	SLE, early nefritis and infectious susceptibility	AR
**C1r/s**	*C1r (NM_001733.7)*	12p13.31				
**C2**	*C2* (GRCh38: 6:31,897,782-31,945,671)	6p21.33				
**C4 a/b**	*C4* (GRCh38: 6:31,982,056-32,002,680)	6p21.33				
**C3**	*C3* (GRCh38: 19:6,677,703-6,720,649)	6p21.33				
**DNASE1L3**	*DNAsi1L3* (NM_004944.4)	3p14.3	DNAsi1L3	Extracellular acid nucleic degradation alteration	SLE, early onset, nephritis, ANCA positive hypocomplementemic urticarial vasculitis syndrome (HUVS)	AR
**DNASE 2**	*DNAsi 2* (GRCh38: 19:12,875,208-12,881,448)	19p13.13	DNAsi 2	Endonuclease dysregulation	SLE antibodies + pancitopenia, membranoproliferative glomerulonephritis, liver fibrosis, deforming arthropathy	AR
**IFIHI**	*MDA5* (NM_022168.4)	2q24.3	melanoma differentiation-associated protein 5	Mutations of type- I IFN Cytosolic sensor for dsRNA	SLE, IgA deficiency, mild lower limb	AD
**TREX1**	*TREX1* (NM_130384.3)	3p21.31	Three Prime Repair Exonuclease 1	Mutations of Type-I IFN degradation of intracellular ds-ss DNA	FCL	AD
**SAMHD1**	*SAMHD1* (NM_080628.3)	20q11.23	SAMHD1	Mutations of Type I IFN cytoplasmic ssRNA/DNA sensor	FCL	AR
**SAVI**	*STING* (NM_198282.4)	5q31.1	Stimulator of interferon gene	Gain-of-function mutations, ↑ type I IFN pathway	Skin vasculopathy, bilateral interstitial lung disease	AD
**PRAAS**	*PSMA3/PSMB8*	14q23.1/6p21.32	Proteasome complex subunit Proteosome chaperone	Loss-of-function mutations in proteasome components, causing type I IFN pathway upregulation	CANDLE syndrome: Chronic Neutrophilic Dermatosis panniculitis with Lipodystrophy, Elevated Temperature	AR
	*PSMB4/PSMB9*	1q21.3/6p21.321q21.3/6p21.32				
	*PSMB4/PSMB8*	6p21.32				
	*PSMB9* (GRCh38: 6:32,854,191-32,859,850)	16q22.1				
	*PSMB10* (GRCh38: 16:67,934,505-67,936,849)	9q33.3				
	*PSMB7* (GRCh38: 9:124,353,464-124,415,441)	14q23.1				
	*PSMA3* (GRCh38: 14:58,244,842-58,272,003)	13q12.3				
	*POMP* (GRCh38: 13:28,659,129-28,678,958)	1				
	*PSMG2* (GRCh38: 18:12,658,737-12,725,739)	8p11.21				
**ISG15 deficiency**	*ISG15* (NM_005101.4)	1p31.33	Interferon-stimulated gene 15 (Stabilizes USP18)		Neurological involvement, mycobacterial susceptibility	AR
**USP18 deficiency**	*UDP18* (NM_019076.4)	2q37.1	UDP18	Altered inhibition of IFNR signalling	Neurological involvement, hepatomegaly, thrombocytopenia	AD
**SMS**	*IFIH1* *DDX58* (NM_014314.4)	2q24.3 9p21.1	IFIH1 DExD/H-Box Helicase 58	Mutations of a cytosolic receptor for dsRNA	Dental and skeletal dysplasia, aortic calcification, glaucome and psoriasis	AD

Abbreviations: AIFEC (autoinflammations and infantile enterocolitis).

**Table 2:** New Eurofever/PRINTO Classification Criteria for FMF.

Presence of pathogenic *MEFV* mutations and at least one among the following: ▪ Duration of episodes 1–3 days ▪ Arthritis ▪ Chest pain ▪ Abdominal pain	OR	Presence of variants of uncertain significance (VUS) of *MEFV* and at least two among the following: ▪ Duration of episodes 1–3 days ▪ Arthritis ▪ Chest pain ▪ Abdominal pain

Abbreviations: PRINTO (Paediatric Rheumatology INternational Trials Organisation).

**Table 3:** New Eurofever/PRINTO Classification Criteria for CAPS.

Presence of pathogenic *NLRP3* mutations and at least one among the following: ▪ Urticarial rash ▪ Red eye (conjunctivitis, uveitis, episcleritis) ▪ Neurosensorial hearing loss	OR	Presence of VUS of *NLRP3* and at least two among the following: ▪ Urticarial rash ▪ Red eye (conjunctivitis, uveitis, episcleritis) ▪ Neurosensorial hearing loss

**Table 4:** New Eurofever/PRINTO Classification Criteria for TRAPS.

Presence of pathogenic *TNFRSF1A* mutations and at least one among the following: ▪ Duration of episodes ≥ 7 days ▪ Myalgia ▪ Migratory rash ▪ Periorbital oedema ▪ Relatives affected	OR	Presence of VUS of *TNFRSF1A* and at least two among the following: ▪ Duration of episodes ≥ 7 days ▪ Myalgia ▪ Migratory rash ▪ Periorbital oedema ▪ Relatives affected

**Table 5:** Inflammasomopathies versus IFNopathies.

INFLAMMASOMOPATHIES	IFNopathies
**CLINICAL SIGNS** Recurrent fever Serositis Arthritis Rash (e.g., urticarial rash) GI symptoms Neurological involvement	**CLINICAL SIGNS** Flu-like fever episodes TORCH-like syndrome without congenital infection Chilblains Raynaud’s phenomenon Panniculitis/lipodystrophy RF positive-JIA resistant to conventional DMARDs Autoimmune SLE—like phenotype Interstitial non-infectious lung disease
**LABORATORY FINDINGS** Elevated inflammatory markers (RCP, ESR, SAA)	**LABORATORY FINDINGS** Normal or slightly increased ESR and CRP with or without leucopeniaFluctuating low-titer ANA and other autoantibodies Leukocytes and IFN increased in cerebrospinal fluid IFN signature

## Data Availability

Not applicable.

## Abbreviations

Systemic autoinflammatory diseases (SAIDs); Gastrointestinal (GI); Central nervous system (CNS); Familial Mediterranean Fever (FMF); Nuclear factor kappa B (NF-κB); Next Generation Sequencing (NGS); Interferon (IFN); Natural killer lymphocytes (NK); Reactive C protein (RCP); Pathogen-associated molecular patterns (PAMPs); Damage-associated molecular patterns (DAMPs); Pattern recognition receptors (PRRs); Toll-like receptors (TLRs); C-type lectin receptors (CLRs); Stimulator of interferon genes (STING); Nucleotide-binding oligomerization domain (NOD)-like receptors (NLRs); Absence in melanoma 2 (AIM2)-like receptors (ALRs); Apoptosis related speck-like protein containing caspase activation and recruitment domains (ASC); Interleukin 1 β (IL-1β); Interleukin-18 (IL-18); Gasdermin-D (GSDMD); N-terminal domain of GSDMD (GSDMD-N); Mitogen-activated protein kinase (MAPK); double-strand DNA (dsDNA); Cyclic GMP-AMP synthase (cGAS); Endoplasmic reticulum (ER); IFN regulatory factors (IRF); RNA helicases retinoic acid-inducible gene I (RIG-I); Melanoma differentiation-associated protein 5 (MDA5); Mitochondrial antiviral signalling (MAVS); IFN receptors (IFNR); Janus kinase/signal transducers and activators of transcription (JAK-STAT); Interferon-stimulated genes (ISGs); Ubiquitin-specific protease 18 (USP18); Sphingosine kinase 2 (SPK2); Macrophage activation syndrome (MAS); NLR Family CARD Domain Containing (NLRC); Autosomal recessive (AR); Ras Homolog Family Member A (RhoA); Autosomal dominant (AD); Pyrin associated autoinflammation with neutrophilic dermatosis (PAAND); Serum amyloid A (SAA); variants of uncertain significance (VUS); Tumor necrosis factor (TNF); Mevalonate kinase deficiency (MKD); Hyper-IgD Syndrome (HIDS); Immunoglobulin D (IgD); Mevalonate kinase (MVK); Mevalonic Aciduria (MA); TNF receptor associated periodic fever syndrome (TRAPS); Phosphatidyl-inositol 3 kinase (PI3K); Pyogenic arthritis, pyoderma gangrenosum and acne (PAPA); Proline-serine-threonine phosphatase interacting protein 1 (PSTPIP1); CD2-binding protein 1 (CD2-BP1); Myeloid-related protein (MRP); Pyoderma gangrenosum, acne and hidradenitis suppurativa (PASH); PSTPIP1-associated myeloid-related-proteinemia inflammatory syndrome (PAMI); WD repeat-containing protein 1 (WDR1); Periodic Fever Immunodeficiency and Thrombocytopenia (PFIT); Hematopoietic stem cell transplantation (HSCT); Reactive oxygen species (ROS); Cryopyrin associated periodic syndromes (CAPS); Cold-induced autoinflammatory syndrome 1 (CIAS1); Familial cold autoinflammatory syndrome (FCAS); Muckle-Wells syndrome (MWS); Chronic infantile neurologic cutaneous articular (CINCA) syndrome/neonatal onset multisystem inflammatory disease (NOMID); Familial cold autoinflammatory syndrome 4 (FCAS4); Familial cold autoinflammatory syndrome 4 (FCAS2); TNF receptor superfamily member 1A (TNFRSF1A); Blau syndrome (BS); Caspase recruitment domain containing protein 15 (CARD15); Receptor-interacting serine/threonine-protein kinase 2 (RIPK2); Leucine-rich repeat (LRR); NOD2 associated AID (NAID); Deubiquitinases (DUBs); Linear ubiquitin chain assembly complex (LUBAC); IL-1 receptor (IL-1R); Heme-oxidized IRP2 ubiquitin ligase 1 L (HOIL-1); SHANK-associated RH domain-interacting protein (SHARPIN); HOIL-1L-interacting protein (HOIP); Macrophage Inflammatory Protein 1α (MIP-1α); Family with Sequence Similarity 105, Member B (FAM105B); Otulin-related autoinflammatory syndrome (ORAS); TNFR-associated factor 6 (TRAF6); A20 haploinsufficiency (HA20); TNFα-induced protein 3 gene (TNFAIP3); Antinuclear Antibodies (ANA); Adenosine deaminase 2 (ADA2) deficiency (DADA2); Cat eye syndrome chromosome region candidate 1 (CERC1); Polyarteritis nodosa (PAN); Deficiency of il-1 receptor antagonist (DIRA); IL1 receptor antagonist protein (IL-1Ra); Deficiency of il-36 receptor antagonist (DITRA); IL-36 receptor antagonist gene (IL36RN); Interferonopathies (IFNopathies); Unfolded protein response (UPR); Aicardi-goutières syndrome (AGS); Toxoplasmosis, rubella, cytomegalovirus and herpes (TORCH); Cerebrospinal fluid (CSF); Ribonuclease H2 subunit B (RNASEH2B); Ribonuclease H2 subunit C (RNASEH2C); Ribonuclease H2 subunit A (RNASEH2A); SAM and HD Domain Containing Deoxynucleoside Triphosphate Triphosphohydrolase 1 (SAMHD1); Adenosine Deaminase Acting on RNA 1 (ADAR1); IFN-induced helicase C domain-containing protein 1 (IFIH1); STING-associated vasculopathy with onset in infancy (SAVI); Transmembrane protein 173 (TMEM173); Anti-neutrophil cytoplasmic antibodies (cANCA); Rheumatoid factor (RF); Juvenile idiopathic arthritis (JIA); Familial chilblain lupus (FCL); Retinal vasculopathy with cerebral leukodystrophy (RVCL); Proteasome associated autoinflammatory syndromes (PRAAS); Proteasome Maturation Protein (POMP); Proteasome assembly chaperone 2 (PSMG2); Joint contractures, muscle atrophy, microcytic anaemia and panniculitis-induced lipodystrophy syndrome (JMP); Chronic Atypical Neutrophilic Dermatosis with lipodystrophy and Elevated temperature syndrome (CANDLE); POMP-related autoinflammation and immune dysregulation disease (PRAID); Nonsense-mediated mRNA decay (NMD); Coatomer complex subunit alpha (COPa); Erythrocyte sedimentation rate (ESR); extractable nuclear antigen (ENA); Lupus anticoagulant (LAC); Beta-2-glycoprotein I (β2GPI); Polymerase chain reaction (PCR); Antiphospholipid antibody syndrome (ALS); Non-steroidal anti-inflammatory drugs (NSAIDs); Recombinant IL-1 receptor antagonist (rhIL-1Ra); IL-1 receptor type I (IL-1RI); Food and Drug Administration (FDA); Magnetic resonance imaging (MRI); European Medicine Agency (EMA); Canakinumab Pivotal Umbrella Study in Three Hereditary Periodic Fevers (CLUSTER); T helper 1 (Th1); complementary DNA (cDNA); Kawasaki Disease (KD); juvenile SLE (JSLE); Inflammatory bowel disease (IBD); Systemic juvenile idiopathic arthritis (sJIA); Psoriatic JIA (pJIA); Juvenile dermatomyositis (JDM); Undifferentiated systemic autoinflammatory diseases (USAIDs); IFN response gene score (IRG-S); IL-18–associated pulmonary alveolar proteinosis and MAS syndrome (IL-18PAP-MAS); NEMO deleted exon 5–autoinflammatory syndrome (NEMO-NDAS); SAMD9L-associated autoinflammatory disease (SAMD9L-SAAD); microRNA (miRNA)
